# Prevalence, Diversity, and Virulence of *Campylobacter* Carried by Migratory Birds at Four Major Habitats in China

**DOI:** 10.3390/pathogens13030230

**Published:** 2024-03-06

**Authors:** Shanrui Wu, Ru Jia, Ying Wang, Jie Li, Yisong Li, Lan Wang, Yani Wang, Chao Liu, Elena M. Jia, Yihua Wang, Guogang Zhang, Jie Liu

**Affiliations:** 1 School of Public Health, Qingdao University, Qingdao 266073, China; 2021024501@qdu.edu.cn (S.W.); ywang0412@qdu.edu.cn (Y.W.); l1998012623@outlook.com (J.L.); liyisong@qdu.edu.cn (Y.L.); wanglan@qdu.edu.cn (L.W.); 2021021085@qdu.edu.cn (Y.W.); 2021021097@qdu.edu.cn (C.L.); 2 Key Laboratory of Biodiversity Conservation of National Forestry and Grassland Administration, Ecology and Nature Conservation Institute, Chinese Academy of Forestry, Beijing 100091, China; jiaru@caf.ac.cn (R.J.); wangyihua2113@163.com (Y.W.); zm7672@126.com (G.Z.); 3 School of Science, Hong Kong University of Science and Technology, Hong Kong 999077, China; emjia@connect.ust.hk

**Keywords:** *Campylobacter*, migratory birds, transmission, molecular epidemiology

## Abstract

*Campylobacter* species, especially *C. jejuni* and *C. coli*, are the main zoonotic bacteria causing human gastroenteritis. A variety of *Campylobacter* species has been reported in wild birds, posing a potential avian–human transmission pathway. Currently, there has been little surveillance data on *Campylobacter* carriage in migratory birds in China. In the current work, fresh fecal droppings from individual migratory birds were collected at four bird wintering/stopover sites in China from May 2020 to March 2021. Nucleic acid was extracted and tested for *Campylobacter* with PCR-based methods. Overall, 73.8% (329/446) of the samples were positive for *Campylobacter,* demonstrating location and bird host specificity. Further speciation revealed the presence of *C. jejuni*, *C. coli*, *C. lari*, *C. volucris*, and an uncharacterized species, which all harbored a variety of virulence factors. Phylogenetic analysis performed on concatenated 16S rRNA-*atpA*-*groEL* genes elucidated their genetic relationship, demonstrating both inter- and intra-species diversity. The wide distribution and high diversity of *Campylobacter* spp. detected in migratory birds in China indicated potential transmission across territories. The existence of virulence factors in all of these species highlighted their public health importance and the necessity of monitoring and controlling *Campylobacter* and other pathogens carried by migratory birds.

## 1. Introduction

As one of the major global causes of diarrheal diseases, *Campylobacter* has been considered the most widespread zoonotic agent of human gastroenteritis in the world [1,2]. *Campylobacter* is also one of the most prevalent pathogens identified from foodborne disease in developed countries, and although epidemiological data are incomplete, *Campylobacter* infection has been detected to varying degrees in developing countries [3,4,5,6]. In its estimate of the worldwide burden of foodborne illnesses, the WHO estimated that foodborne *Campylobacter* spp. caused more than 95 million illnesses, 21,374 deaths, and nearly 2,142,000 DALYs^10^ in 2010 [7,8]. As is well known, the most prevalent species associated with campylobacteriosis are *Campylobacter jejuni* and *Campylobacter coli*. Notably, other *Campylobacter* species of clinical significance have been identified, including *Campylobacter concisus*, *Campylobacter lari*, *Campylobacter upsaliensis* [6], and *Campylobacter ureolyticus* [9,10]. In addition to common gastroenteritis, *Campylobacter* is also associated with a number of immunoreactive complications, such as *Guillain-Barre syndrome* and *Miller-Fisher syndrome*, as well as brain abscess, meningitis, bacteremia, septicemia, endocarditis, myocarditis, reactive arthritis, and clinical manifestations leading to reproductive tract complications, which often occur in immunocompromised people, such as the elderly and children [11,12,13].The important and extensive clinical significance of *Campylobacter* is being increasingly recognized.

Birds, especially migratory birds, have long played an important role in the spatial transmission of zoonosis diseases, e.g., avian flu [14]. Birds can indirectly spread pathogens via feces, by contaminating water, and by carrying ticks, etc. [15,16,17,18,19]. As a typical zoonotic bacterial pathogen, *Campylobacter* is widely detected and researched in wild and domestic animals, particularly poultry and livestock, such as chicken, pigs, cattle, sheep, and the corresponding food products [20,21,22,23]. A variety of migratory birds have also been found to be excellent vectors and reservoirs of *Campylobacter* [24,25,26]. There are nine migratory bird migration routes in the world, three of which pass through China. In other words, most of the land in China is on one of these important global bird migration routes [27]. However, little is known about pathogen carriage in the migratory birds in China.

Xizang, Qinghai, Heilongjiang, and Hebei are part of the most important breeding and stopover grounds for many migratory birds in China. In the current work, we investigated the prevalence and diversity of *Campylobacter* species in these regions and further characterized their virulence and genetic traits.

## 2. Materials and Methods

### 2.1. Sample Collection

A total of 446 fresh fecal dropping samples from migratory birds were collected at four bird wintering/stopover sites in China, namely Xingkai Lake in Heilongjiang, Cangzhou in Hebei, Longbao Reserve in Qinghai, and counties along the Yarlung Tsangpo River in Xizang from May 2020 to March 2021, preserved in the transport medium (0.9% NaCl, 0.2 g/L penicillin, 2 g/L streptomycin sulfate, 20% glycerol), and stored at −80 °C until testing. 

### 2.2. Total Nucleic Acid Extraction

Nucleic acid was extracted with a Viral DNA/RNA Extraction kit (Tianlong Technology Co., LTD, Xi’an, China) or QIAamp Fast DNA Stool Mini Kit (Qiagen, Hilden, Germany) following the manufacturer’s instructions, with pretreatment as described previously [28]. External controls (Phocine herpesvirus and MS2 bacteriophage) were spiked into each sample to monitor the extraction and amplification efficiency. One extraction blank was included per batch of extractions to rule out laboratory contamination.

### 2.3. Detection and Speciation of Campylobacter *spp.*

PCR assays were adapted or modified from publications as appropriate or developed and validated as needed (Supplemental Table S1, with references and indications if the assay was modified or newly designed). TaqMan-probe-based real-time PCR targeting 23S rRNA and conventional 16S PCR followed by gel electrophoresis were used for detection of the *Campylobacter* genus [29]. Speciation was performed via PCR amplicon sequencing of 16S, *atpA*, and *groEL* (also known as *cpn60*) [30,31]. *C. jejuni* and *C. coli* were differentiated and quantified with species-specific qPCR targeting *hipO* and *glyA*, respectively [28].

The TaqMan probe-based PCR reaction contained 2× AgPath buffer (AgPath-ID^TM^ one step RT-PCR kit, Thermo Fisher Scientific, Carlsbad, CA 92008, USA), 25× AgPath Enzyme mix, primers and probes at final concentrations of 900 nM and 250 nM, respectively, and template DNA. The qPCR cycling condition was set as 95 °C for 10 min, 45 cycles at 95 °C for 15 s, and 60 °C for 1 min.

The conventional PCR reaction included 2× Taq Plus Mater mix II (Vazyme, Nanjing, China), 400 nM forward and reverse primers, DNA template, and nuclease-free water. PCR was performed with the following conditions: initial denaturation at 95 °C for 3 min, 45 cycles at 95 °C for 15 s, 55~60 °C for 20 s (annealing temperature varied by target, see Table S1), and 72 °C for 60 s, and a final extension at 72 °C for 10 min. The PCR products were examined via gel electrophoresis, followed by amplicon sequencing (Sangon, Qingdao, China).

### 2.4. Detection of Anti-Microbial Resistance (AMR)

Samples positive for *Campylobacter* were tested for two *Campylobacter* specific ARGs with qPCR, i.e., *gyrA* T86I for *C. jejuni* resistance to fluoroquinolone and 23S A2075G for macrolide resistance in *Campylobacter* species (Supplemental Table S1) [29]. The qPCR conditions were the same as above.

### 2.5. Detection of Virulence Genes

The Virulence Factor Database (VFDB) was used to determine the consensus virulence genes among the different *Campylobacter* species, including *C. jejuni*, *C. coli*, *C. lari*, and *C. volucris*. Species-specific primers were designed or modified from publications [28,32] (Table S1). Seven known virulence genes (*cdtA*, *cdtB*, *cdtC*, *cadF*, *ciaB*, *cheY*, *flaA*) associated with *Campylobacter* toxin, adherence, invasion, and motility, respectively, were tested via PCR on *C. jejuni*, *C. coli*, *C. lari*, and *C. volucris* positives. VFDB was also used to predict the putative virulence-associated genes of the strain RM12651 (NCBI accession number CP059600), with an e-value cutoff set at 1 × 10^−5^, identity at 40%, and the query coverage at 50%. The genes identified were further compared with those from four *Campylobacter* species (*C. jejuni*—genome assembly: ASM1336377v1, ASM2434966v1; *C. coli*—genome assembly: ASM973039v1, ASM148384v1; *C. lari*—genome assembly: ASM824502v1, ASM1920v1, ASM81640v1; *C. volucris*—genome assembly: ASM434504V1, ASM824504v1) and classified into two categories, i.e., common among the 5 species or unique to RM12651. Four genes from the common set and three from the unique set were selected for PCR primer design and testing, with the same conditions as above.

### 2.6. Identification of Migratory Bird Types

The bird types observed were recorded during fecal dropping collection. For precise identification, the mitochondrial cytochrome oxidase subunit I gene (COI) was amplified with the primers specifically designed for birds [33] with modifications to expand the detection scope to accommodate the bird types in the current study. The amplicons were sequenced.

### 2.7. Statistical Analyses

A chi-square test or Fisher’s exact probability method was performed to compare the prevalence of *Campylobacter* detection and the presence of virulence genes between regions or migratory birds. SPSS software, version 26.0, was used for the analysis. Two-tailed *p* values were calculated, and values of 0.05 were considered statistically significant.

## 3. Results

### 3.1. Migratory Bird Types Included in the Current Study

Based on the observation during the sample collection, the bird populations in Heilongjiang, Xizang, and Qinghai were relatively unitary, i.e., predominantly *Anser albifrons* or *Anser fabalis* in Heilongjiang and *Anser indicus* in Qinghai and Xizang. A random selection of 146 samples was tested with COI PCR, and the sequencing results confirmed the previously recorded bird types. Hebei, on the other hand, had diverse species of migratory birds exclusively requiring COI determination. The migratory bird types identified included Anatidae, Laridae, Scolopacidae, Charadriidae, Recurvirostridae, Phalacrocoracidae, etc. (Table S2). 

### 3.2. Prevalence of Campylobacter in Different Regions and Birds

A sample was considered *Campylobacter*-positive when either 23S rRNA qPCR was positive or the 16S rRNA amplicon yielded correct sequences. *Campylobacter* spp. was detected in 73.8% (329/446) of the fecal dropping samples. The detection in Heilongjiang (41/43, 95.3%) and Hebei (80/87, 92.0%) was higher than that in Xizang (171/266, 64.3%, *p* < 0.008) and Qinghai (37/50, 74.0%, *p* < 0.008). A subset of 168 samples was further subjected to speciation. As shown in Table 1, five species, including *C. jejuni* (22, 13.1%), *C. coli* (3, 1.8%), *C. lari* (13, 7.7%), *C. volucris* (5, 3.0%), and an unknown *Campylobacter* species (94, 56.0%), showing 100% identity to RM12651 (NCBI accession number CP059600), were identified with the combination of 16S rRNA, *atpA*, and *groEL* amplicon sequencing. Species-specific qPCRs for *C. jejuni* and *C. coli* were performed to further differentiate the two species and quantify the bacterial load of *C. jejuni* and *C. coli* to be in the magnitude of 10^6^ and 10^5^ per gram of fecal droppings, respectively. In addition, 20.8% (35/168) of the samples were not speciated or indistinguishable among *C. novaezeelandiae*, *C. armoricus*, *C. peloridis*, and *C. volucris*. *C. jejuni*/*C. coli* was mostly detected in Xizang (22/25), except for one *C. jejuni* isolate in Heilongjiang and two in Hebei. *C. lari* (10/13) and *C. volucris* (4/5) were mainly detected in Hebei. 16S rRNA, *atpA*, and *groEL* sequencing results all indicated an unknown *Campylobacter* species, matching the sequences of NCBI accession number CP059600 through BLAST. This unknown species has been reported in North American regions and was found to be the predominant *Campylobacter* in *Anser fabalis/Anser albifrons* of Heilongjiang and *Anser indicus* of Xizang and Qinghai in the current study (Table 1).

### 3.3. Phylogenetic Analysis across Campylobacter Species in the Four Habitats

**Figure 1:**
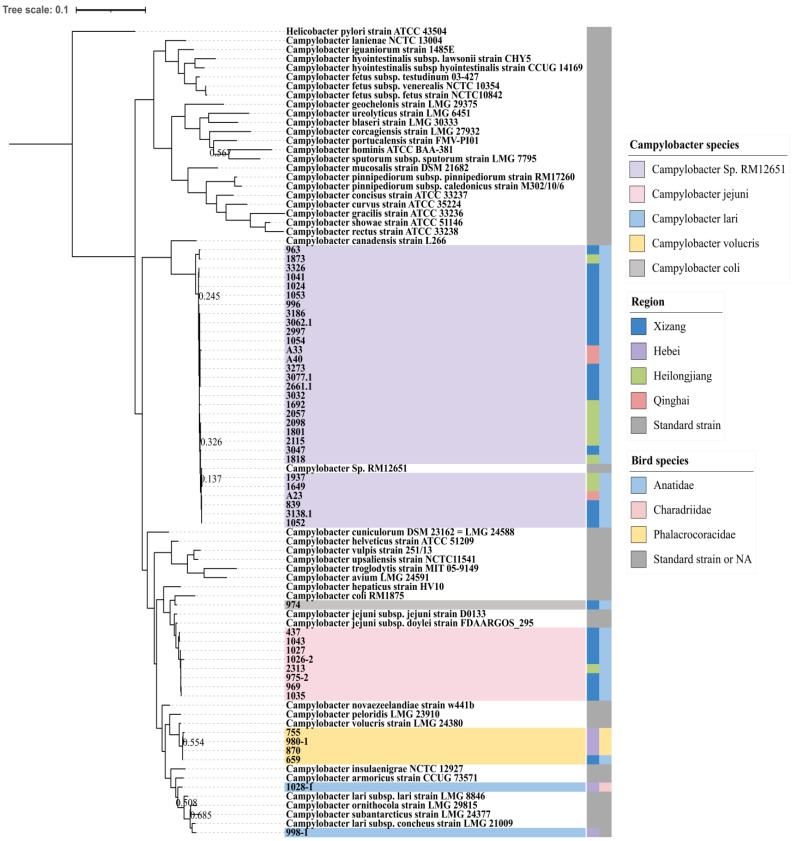
Phylogenetic analysis based on the 16S rRNA—*atpA—groEL* gene in different habitats and migratory birds. Fasttree was used to construct the tree. *Helicobacter* was used as an outgroup. The figure was prepared with iTOL (Interactive Tree of Life). Different colors in the two columns represent different regions and birds, respectively. The Bootstrap value is displayed only when it was <0.7.

Phylogenetic analysis based on concatenated 16S rRNA—*atpA—groEL* genes demonstrated diversity across *Campylobacter* species at different habitats. Based on the similarity to known species, the samples were divided into five groups, i.e., *C. canadensis*, *C. jejuni*, *C. coli*, *C. volucris*, and *C. lari*. Most of the *Campylobacter* identified in this study formed a distinct cluster, showing sequence similarity to RM12651 (NCBI accession number CP059600), which was closely related to *C. canadensis*. The unspeciated samples were grouped into one cluster separated from *Campylobacter* on the phylogenetic tree based on 16S alone (Figure S1). The sample No. 974, positive for the *glyA* gene, was more closely related to *Campylobacter jejuni* on the 16S phylogenetic tree but turned out to be clustered with *Campylobacter coli* based on the concatenated 16S rRNA—*atpA—groEL* gene (Figure 1 and Figure S1), which apparently improved the differentiation among species.

### 3.4. Detection of Virulence Genes in Known Campylobacter Species

**Figure 2:**
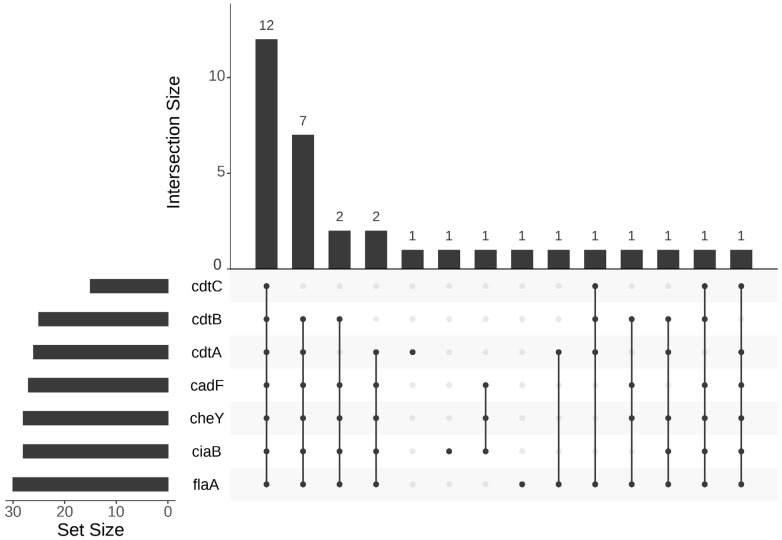
Upset of *Campylobacter* (*C. jejuni*, *C. coli*, *C. lari*, *C. volucris*) with respect to the detection of different virulence genes.

Species-specific primers were designed for the interrogated virulence factors. The specificity of the assays was confirmed via PCR amplicon sequencing. All the samples carried at least one of the seven virulence genes, while all seven virulence genes were detected in at least one sample of the four *Campylobacter* species, i.e., *C. jejuni*, *C. coli*, *C. lari*, and *C. volucris* (Table S2; Figure 2). Of the 14 combinations (Figure 2), 36.4% (12/33) of the specimens possessed all seven virulence factors, six of which were positive for *C. lari* from Hebei. Among *C. jejuni* positives, the *Campylobacter* flagella motility factor genes *flaA* and *cheY* were positive in 14 (93.3%) and 13 (86.7%) samples, respectively. For toxin genes, 76.5% of *C. jejuni* positives tested positive for *cdtA* and *cdtB*, while only 23.5% were positive for *cdtC*. The adherence gene *cadF* and invasion gene *ciaB* were possessed by 70.6% and 80.0% of *C. jejuni*. Two of three *C. coli* tested positive for *cdtA*, *cdtB*, *cheY*, and *flaA*, while one was positive for *cdtC* and *cadF*. All *C. coli* positive samples tested positive for *ciaB*. The genes *cdtA*, *cdtB*, *cdtC*, *cadF*, *ciaB*, *cheY*, and *flaA* were detected in more than 81% of *C. lari* samples, while all carried *flaA*. Moreover, 50% of *C. volucris* samples were positive for *cdtB* and *cdtC*, 75.0% for *cdtA* and *flaA*, and 100.0% for *cadF*, *ciaB*, and *cheY* (Table S3). 

Only one (4.5%, 1/22) of the *C. jejuni* positive samples was found to carry a *gyrA* 86I mutation, associated with fluoroquinolone resistance, and both were from Xizang. No 23S 2075G mutation associated with macrolide resistance was detected (*n* = 92).

### 3.5. Putative Virulence Determinants of Campylobacter *sp.* RM12651-like Samples

**Figure 3:**
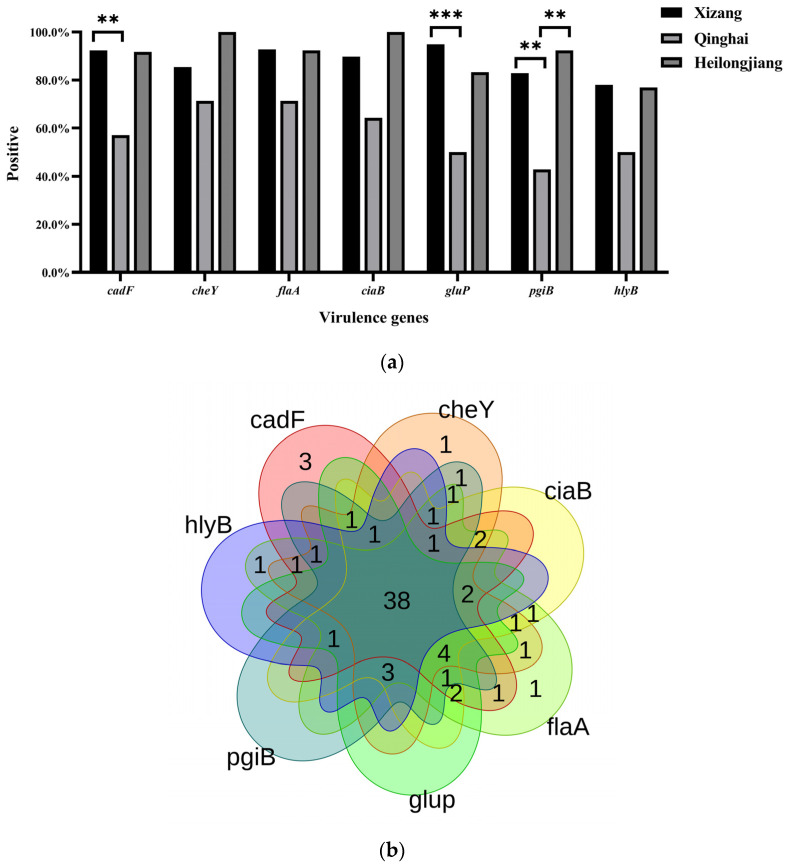
Virulence genes detected in RM12651-like *Campylobacter* sp. (**a**) Prevalence of virulence-associated genes predicted from the *Campylobacter* Sp. RM12651 genome at the three sites. ** means *p* < 0.05; *** means *p* < 0.005. Hebei was not shown due to the small sample size of RM12651-like *Campylobacter*. (**b**) Venn diagram of the distribution of the virulence genes.

One hundred and forty-two genes in the genome of *Campylobacter* sp. RM12651 met the criteria based on VFDB, involved in exotoxin, adhesion, invasion, motility, LOS synthesis, and LpS synthesis (Table S4). It was worth noting that cytolethal distending toxin (CDT) was not found in the genome of RM12651. Compared to *C. jejuni*, *C. coli*, *C. lari*, and *C. voluvris*, 129 genes were common to the five species, while 13 genes were unique to the RM12651 genome. PCRs targeting the consensus virulence genes, i.e., *cadF*, *ciaB*, *cheY*, *flaA*, and three uniquely identified in RM12651, i.e., *gluP*, *hlyB*, and *pgiB*, were developed and tested on RM12651-like samples (Tables S4–S6). All 73 samples tested carried at least one of these genes (Figure 3). The detection rates of the seven genes were all above 72.0%, with *cdaF*, *gluP*, and *pgiB* in Xizang significantly higher than those in Qinghai (*p* < 0.016) possessing the same migratory bird type. The prevalence of *pgiB* in Qinghai was also lower than in Heilongjiang (Figure 3a, *p* < 0.016). As shown with the Venn diagrams of Figure 3b, most (38/73, 52.1%) of the samples were positive for all seven genes, covering all four areas of this study (Figure 3b)

## 4. Discussion

This study evaluated the prevalence, diversity, and virulence of *Campylobacter* from migratory birds in the habitats in Xizang, Qinghai, Heilongjiang, and Hebei. *Campylobacter* was widely detected in migratory birds in these regions, including the known pathogenic species (*C. jejuni*, *C. coli*, *C. lari*, and *C. volucris*), which demonstrated a location- or host-specific distribution. *C. jejuni* and *C. coli* were mainly found in *Anser indicus* in Xizang at 7.1% (19/266) and 1.1 (3/266) detection rates, respectively, while *C. lari* and *C. volucris* were mainly in Hebei at 21.3% (10/47) and 8.5% (4/47), respectively. *C. lari* was detected exclusively from *Charadriiformes*, including *Chroicocephalus ridibundus*, *Numenius arquata*, and *Charadrius alexandrinus*. Three of the five cases of *C. volucris* were from *Phalacrocorax carbo*, with the remaining two unknown. Due to the availability of the samples, 40.8% (182/446) samples were not tested further for 16S rRNA, which most likely underestimated the detection of *Campylobacter* and *Campylobacter* species. The overall *Campylobacter* prevalence in our study was higher than in other reports on wild birds from Beijing, China (11.0%), South Korea (6.0%), Santa Fe, Argentina (24.0%), and Sandhill Crane at the Central Platte River, USA (48.0%) [24,26,34,35]. However, these studies mostly focused on *C. jejuni*, *C. coli*, and *C. lari*, for which detection in our study was relatively lower.

RM12651-like *Campylobacter* sp. appeared to be the predominant *Campylobacter* species in the *Anser* genus. The detection rate of *Campylobacter* spp. in Heilongjiang (21/43, *p* < 0.008) was significantly higher than that in Xizang (50/266), Qinghai (14/50), and Hebei (8/87). On one hand, this reflected the differential distribution in bird species. Heilongjiang is mainly dominated by *Anser albifrons*/*fabalis*, whereas Xizang and Qinghai are dominated by *Anser indicus*, and Hebei, with a variety of birds, is dominated by *Charadriiformes*. On the other hand, this revealed the impact of the geographic location of Heilongjiang. Xingkai Lake is located in the center of the East Asian–Australian Migration Route, which enables increased contact with migratory birds from the United States, Canada, or other areas. This *Campylobacter* species was originally isolated from fecal samples of Canada geese in California, USA. Phylogenetic analyses of putative new taxa based on the *atpA* allele showed that 10 unknown isolates, including strain RM12651, clustered into a single branch, indicating the likelihood of a new *Campylobacter* species [24,36]. Our samples were clustered with RM12651 on the 16S rRNA, *atpA*, and *groEL* phylogenetic trees, which was most closely related to *Campylobacter canadensis* but separated from other known species. To our knowledge, this is the first report on RM12651-like *Campylobacter* outside of the Americas. A further genome-wide analysis is required to demonstrate the possible transmission pathways across continents and the evolutionary relationships. As little has been known about *Campylobacter* carriage in the migratory birds in China, interestingly but not surprisingly, a separate group of *Campylobacter* was also identified in the current work, indicating another potential emerging species.

The presence of virulence factors has been widely used to evaluate bacterial pathogenicity. At least one of the seven assayed virulence genes was carried by the *Campylobacter* positives interrogated. Cytolethal distending toxin (CDT), produced by *Campylobacter*, can cause the arrest of different cells, where its toxicity needs the complex of *cdtA*, *cdtB*, and *cdtC* [12]. All three toxin genes were detected in 36.4% (12/33) of the samples, with *cdtC* as the main limiting factor. Despite the fact that CDT was not predicted in the RM12651 genome, *hlyB*, a toxin-related gene with 45.8% identity to *Escherichia coli*, was detected in 72.6% of RM12651-like samples. *CadF* and *flpA*, two microbial surface components recognizing adhesive matrix molecules (MSCRAMMs), have been shown to be independently required to adhere to fibronectin and further deliver the *Campylobacter* infection antigen (Cia) effector proteins, which in turn are required to invade the host cell [37,38]. A high percentage (84.3%, 59/70) of the co-existence of *ciaB* and *cadF* genes was detected, including in the samples positive for RM12651-like *Campylobacter*. The connection among the motility, chemotaxis, and virulence of *C. jejuni* has long been appreciated, such as *flaA* and *cheY* [38,39]. The detection rate for these two genes was over 86%. Further work is needed to verify their pathogenicity. 

The impact of bird migration on the transmission of pathogens has been extensively studies, mostly focusing on Avian Flu [40]. For *Campylobacter*, either wild birds or domestic poultry may serve as reservoirs through continuous propagation and toleration, with the absence of clinical signs of infection. *Anser indicus* mainly forages in farmland, mixed with black-necked cranes (*Grus nigricollis*) and Ruddy Shelduck (*Tadorna ferruginea*), where they feed on *Poaceae Barnhart*, such as the grains left after harvest, like barley and wheat [41]. According to satellite transmitters, *Anser indicus* flies south from Mongolia and Qinghai to spend the winter in the Yarlung Zangbo River basin of Xizang and India every autumn [42]. As an important corridor in the migration zone of the East Asian–Australian migratory bird migration route, Xingkai Lake is an essential stopover breeding site for cranes, Anatidae, such as *Anser albifrons* and *Anser fabalis*, and shorebirds [43,44]. Carex and rice sprouts from meadows, paddy fields, and mudflats are the main food source of *Anser albifrons* [45]. *Anser albifrons* and *Anser fabalis* tend to overwinter at Poyang Lake in Jiangxi and travel to Russia via Heilongjiang to breed. In contrast, gulls have a more diverse habitat, including the ocean, and often congregate at sites, such as landfills, to forage, with fish and insects as their main food source [46]. *Chroicocephalus ridibundus*, the main species in Hebei, usually departs from Yunnan every year and travels via Hebei to Russia to breed. Migratory birds can thus serve as asymptomatic vectors for the transmission of *Campylobacter*, as well as other pathogenic microorganisms. The existence of different birds, including poultry, and animals, either wild or domestic, in the same space, or sharing the same contaminated water source and other environmental elements may increase the chance of mutual infection and spreading. For humans, the highest risk of campylobacteriosis has been proven to come from the consumption of contaminated meat from chicken and other poultry [9]. Additionally, direct contact with animals, most likely domestic animals, or other fecal–oral routes can lead to the acquisition of *Campylobacter*. Humans also have a low infection dose of *Campylobacter*, at about 500. In the current study, the bacterial loads of *C. jejuni* and *C. coli* were quantified with qPCR to be 10^6^ and 10^5^ per gram of fecal droppings, respectively. Therefore, it is not surprising that exposure to contaminated wild bird droppings in playgrounds has been identified as a new environmental source of campylobacteriosis, especially for children [47]. The active long-term surveillance of *Campylobacter* in wild and domestic animals and the relevant environment is of great importance to understand the transmission pathways and to further provide guidance on cutting down the potential transmission from the wild sources to farms or directly to humans.

Based on the preliminary phylogenetic analysis (Figure 1), *Campylobacter jejuni* identified in this study clustered with the known isolate from a human source, while all the other species seemed to be closely related to the strains isolated from animal sources. A more vigorous sequence comparison is required to characterize their genetic relationships. Meanwhile, concerning the high prevalence and wide distribution of these *Campylobacter* species, particularly the uncharacterized RM12651-like species, surveillance in people with potential exposure would be of great significance for determine the clinical or public health relevance. With high sensitivity and specificity, multiplex real-time PCR can be developed based on the current findings and utilized for the quick screening of these *Campylobacter* species in humans, domestic animals, and the environment.

The main limitation of the current study is that bacterial culture was not feasible because of the use of antibiotics during sample collection and storage as the standard procedure for avian flu studies. Therefore, similar to a large number of studies using molecular methods, which do not discriminate the viability of the pathogens, PCR detection alone may overestimate the potential health risk. Moreover, this has hindered the genomic analysis of the uncharacterized RM12651-like species. Instead, metagenomic sequencing is ongoing with the intention to confirm its relationship with the RM12651 strain. The lack of isolates also prevented us from characterizing the antimicrobial resistance, as it has been cumbersome to interpret the detection of ARGs in complexed specimens [29]. However, we were able to interrogate the *Campylobacter*-specific AMR targets associated with fluoroquinolone and macrolide resistance, respectively. Fluoroquinolones, aminoglycosides, and macrolides are the most commonly used drugs for the treatment of human campylobacteriosis. These antibiotics are also frequently applied to food animals, such as poultry, to control bacterial infections on farms to improve the animals’ growth [32,48,49]. The potential role of migratory birds in the transfer of antibiotic resistance genes and antibiotic-resistant bacteria has gradually attracted attention [50,51,52,53], including drug resistant *Campylobacter*. Another limitation was that temporal variation and seasonality of *Campylobacter* carriage by migratory birds were not evaluated due to the availability of the samples. Studies have shown the detection of certain pathogens associated with the seasonal migration of wild birds or animals, such as highly pathogenic avian influenza H5N8 [40] and Kyasanur forest disease virus [54]. Such information would improve the targeted prevention and control of diseases.

A further assessment and investigation of health risks resulting from the carriage of *Campylobacter*, in particular emerging species, and other pathogens by migratory birds is needed. Once confirmed, appropriate measures, such as plant-mediated biofilters [55], may be taken to attenuate these bacteria in the corresponding environments with a one health approach.

## Figures and Tables

**Table 1:** Distribution of *Campylobacter* species at the four habitat sites.

*Campylobacter* Species	Detection Rate	16S Identity	Region			
			Xizang (n = 82)	Qinghai (n = 14)	Heilongjiang (n = 25)	Hebei (n = 47)
*Campylobacter* sp. (RM12651)	94 (56.0%)	100.0 (100.0, 100.0)	51	14	21	8
*C. jejuni* ^#^	22 (13.1%)	100.0 (99.8–100.0)	19	-	1	2
*C. coli* ^#^	3 (1.8%)	100.0 (NA)	3	-	-	-
*C. lari*	13 (7.7%)	100.0 (99.8–100.0)	3	-	-	10
*C. volucris*	5 (3.0%)	100.0 (99.8–100.0)	1	-	-	4
*C. novaezeelandiae/armoricus/peloridis/volucris* *	8 (4.8%)	100.0 (99.7–100.0)	-	-	-	8
Uncultured bacterium clone *	17 (10.1%)	99.6 (97.3–100.0)	4			13
Others	10 (6.0%)	97.5 (96.6–98.2)	5		3	2

* Indistinguishable with 16S rRNA, *atpA*, or *groEL* sequencing. ^#^ One *C. coli* isolate was mixed with *Campylobacter* sp. (RM12651); three *C. jejuni* isolates were mixed with *Campylobacter* sp. (RM12651). NA: Not applicable.

## Data Availability

The sequences generated and presented in this study have been deposited in NCBI GenBank, with the accession numbers PP333242 through PP333389 assigned. The information for the sequences used for virulence factor prediction is presented in the Supplementary Material. The raw data are available upon request from the corresponding author.

## Supplementary Materials

The following supporting information can be downloaded at: https://www.mdpi.com/article/10.3390/pathogens13030230/s1, Table S1: Primer sequences used in this study [2,29,31,32,36,56,57]; Table S2: Distribution of *Campylobacter* detected in different migratory bird species at the four habitats; Table S3: Detection of virulence genes in the five *Campylobacter* species; Table S4: Presumptive RM12651 virulence genes identified by BLAST analysis of protein sequences. Subject sequences are predominantly from *C. jejuni*, *C. coli*, *C. lari*, and *C. volucris* (see Materials and Methods); Table S5: Presumptive RM12651 virulence genes shared with *C. jejuni*, *C. coli*, *C. lari*, and *C. volucris*; Table S6: Presumptive RM12651 virulence genes unique to *C. jejuni*, *C. coli*, *C. lari*, and *C. volucris*; Figure S1: Phylogenetic analysis based on the 16S rRNA gene in different habitats and migratory birds.
