# Supplementary material for: Prevalence, Diversity, and Virulence of Campylobacter Carried by Migratory Birds at Four Major Habitats in China
Source: Pathogens. 2024 Mar 6;13(3):230. doi: 10.3390/pathogens13030230 (PMC10975922; doi:10.3390/pathogens13030230)
Supplement: Supplementary file 1 [file pathogens-13-00230-s001.zip › CampySupplementalTable1-3-Fig1.pdf]

Supplemental materials

Table S1. Primer and probe sequences used in this study.

Purpose	Target		Primer sequence	Detection	Reference[a]	Annealing temperature (°C)	Amplicon Size (bp)
Genus detection	16S	forward	GGATGACACTTTTCGGAGC	Genus	Modified, D Linton[1]	55	800
		reverse 1	CATTGTAGCACGTGTGTC				
		reverse 2	CATTGTAGCACGTGTGTA				
	23S	forward	GATCCAGTGAAATTGTAGTGGAGGT	Genus	Pholwat, S.[2]	60	158
		reverse	GGCTCATATACAACTGGCGTCATA				
		probe	GACGGARAGACCC				
Speciation and phylogenetic analysis	*atpA*	forward	ATGGACTTAAGAATATTATGGC	*C. jejuni*/*C. coli*	Modified, Dingle[3]	50	1300
		reverse 1	ATAAATTCCATCTTCAAATTCC				
		reverse 2	CACGYTCAAGTTGTTTTCTACT(sequence)				
		forward	GARCCAATTGAYGCTAAAGG	*C. lari*	Miller[4]	53	924
		reverse	TTTAADAVYTCAACCATTCTTTGTCC				
		forward	GGTATGGCHTTAAACCTTGAAGA	*C.* Sp (RM12651)	This study	52	1060
		reverse	TGACCACGCTCWARTTGTTTTCT				
		forward	GGTGATGCTTTAATTGGTCGTG	*C. volucris*	This study	60	1091
		reverse	AACGGATACAATGCTGCTTCA				
	cpn60	forward	GATCAAGCAGGCGATGGAAC	*C. jejuni*	Hill, J. E[5]	60	614
		reverse	GCTTTTCTTCTATCGCCAAAACCTG				
		forward	CTTGAAAATATGGGYGCTTCAC	*C. coli*	This study	58	665
		reverse	GCTTTTCTTCTATCKCCAAAACCT				
		forward	GAAAATATGGGTGCTTCTTTRGT	*C. lari*	This study	56	707
		reverse	GAAATCACTTCACCACCTGTT				
		forward	CAAGCAGGAGATGGAACTACAAC	*C.* Sp (RM12651)	This study	60	651
		reverse	CGTTCCACCTGTTAAGATTGC				
		forward	AAGCWGGTGATGGAACAACAAC	*C.ornithocola*/	This study	60	655
		reverse	GAAATCACTTCGCCACCTGT	*C. subantarcticus*			
		forward	CTGATCAAGCAGGAGATGGA	*C. volucris*	This study	59	680
		reverse	TGTTCTTCCAAGCTCTTCTGAG				
	hipO	forward	CTTGCGGTCATGATGGACATAC	*C. jejuni*	Liu, J.[6]	60	122
		reverse	AGCACCACCCAAACCCTCTTCA				
		probe	TGCTTGCTGCAAAGTATT				
	glyA	forward	AAACCAAAGCTTATCGTGTGC	*C. coli*	Liu, J.[6]	60	125
		reverse	AGTGCAGCAATGTGTGCAAT				
		probe	TAAGCTCCAACTTCATCCG				

Virulence	*cdtA*	forward	ATCGTACCTCTCCTTGGCG	*C. jejuni*	Poudel, S.[7]	60	440
		reverse	CGGAGCAGCTTTAACGGTTTG				
		forward	GCCAAGGCTAAAATCTCCAAA	*C. coli*	This study	60	192
		reverse	TGCCAAGCTCTTGCATCTC				
		forward 1	CGGTTTGGGCTTTAAATCCA	*C. lari*	This study	58	388
		reverse 1	GCACTAATGCTCCATTGTCTAT				
		forward 2	CAGTTTGGGCCTTAAATCCA				
		reverse 2	GCGCTAACACTCCATTGTCTAT				
		forward	TGGGCGATATGTATGATGATG	*C. volucris*	This study	60	480
		reverse	TCCAAAATTGAGCTTGATTGC				
	cdtB	forward	TGGAGGAACAGATGTAGGAGC	*C. jejuni*	Poudel, S.[7]	60	180
		reverse	GCTTGAGTTGCGCTAGTTGG				
		forward	CTAGYAAGTGGGGGAAATGA	*C. coli*	This study	60	183
		reverse	CGTAGAAGAAGGCGGAACAAC				
		forward	AGAGTDGATGTTGGRGCAAAT	*C. lari*	This study	60	304
		reverse	TACGCGMTCTTAAATCHGSATCA				
		forward	AAAATCACCTGCAACCATCC	*C. volucris*	This study	60	464
		reverse	CTGCAACCACAGAAAGCAAA				
	cdtC	forward	GCTCCAAAGGTTCCATCTTCTAAG	*C. jejuni*	This study	60	269
		reverse	GCCTTTGCAACTCCTACTGG				
		forward	GCCTAGCTTGGATGAATTAG	*C. coli*	This study	60	411
		reverse	TCTATGGCGATACTAGAGTCAG				
		forward	GCAGTTTATAAAYCCTAGAGGTGCTG	*C. lari*	This study	60	315
		reverse	GGAGTTGCTTCAACRATAGCAG				
		forward	TCTAAACCGAAACGCTGCTC	*C. volucris*	This study	60	430
		reverse	GCGTTTGCTGAGATGGAATC				
	cadF	forward	CTGCTAAACCATAGAAATAAAATTTCTCAC	*C. jejuni*/*C. coli*	Liu, J. [6]	60	221
		reverse	CTTTGAAGGTAATTTAGATATGGATAATCG				
		forward	GCTCAATATGGTGCRGGTTT	*C. lari*	This study	60	259
		reverse	CACAACCTATATGATCAAGCAAAGC				
		forward	TGGTTATGAGCACCAAAAAGG	*C.* Sp (RM12651)	This study	60	674
		reverse	TGTCTTCGCTAACACCAGCTT				
		forward	GTGGTATGTTTGCCCAATATGGT	*C. volucris*	This study	60	249
		reverse	AGGCACCTTTTCTTGGTTCTG				
	flaA	forward	ATGCTTCAGGGATGGCGATA	*C. jejuni*/*C. coli*	This study	60	178
		reverse	CCATCTTGAGCCGCTTGAGT				
		Forward 2	GATGATGCTTCKGGKATGGCTA	*C. lari*	This study	59	192
		reverse 2	AGTRCTTTGWCCATCTTGWGC				
		forward	TAACTCTGCTGCTGATGATGCT	*C.* Sp (RM12651)	This study	60	229
		reverse	AGCTTGAAGTGAGCGTCTTGTT				
		forward	AGATGATGCTTCAGGTATGGCTA	*C. volucris*	This study	60	193

	reverse	AGTGCTTTGCCCATCTTGAG				
	forward 1	ATGCTTCAGGTATGGCTATTG	*C. volucris*	This study	56	418
	reverse 1	CTACTTTTAGCTTGAGATCCTGTT				
cheY	forward	TGATGACAGTTCTACTATGAGAAGG	*C. jejuni*	This study	56	362
	reverse	CTTCTCCACTTCCTGTTCCT				
	forward	TGATGACAGTTCTACTATGAGAAGG	*C. coli*	This study	56	283
	reverse	CAGTAATCACCTCAGCCTTG				
	forward	GTAGTTGATGATAGTTCTACCATGAG	*C. lari*	This study	56	248
	reverse	CCACCTTCTGTWGTTACCATGA				
	forward	CAAAGATTAGGTCATGAGGATG	*C.* Sp (RM12651)	This study	56	210
	reverse	TTCTGCTTTACCACCTTCAG				
	forward	GATGTTTTAGAGGCTGAGCA	*C. volucris*	This study	56	198
	reverse	TTCAGCTTTACCACCTTCTG				
CiaB	forward	AGCTGAATTTGCTTGGATGA	*C. jejuni*	This study	58	279
	reverse	CCATAAAATATCATCGGAGTGC				
	forward	GGCTGAATTTGCATGGATGA	*C. coli*	This study	60	306
	reverse	GCAGAAAACAAACCCTTAAGCTC				
	forward	GTTCAAGGTGTGCATAATATAGGTGT	*C. lari*	This study	58	704
	reverse	CCCTTAAGCTCTGCYCCATAA				
	forward	AGACTTCATTCAAGTTGGGCATT	*C.* Sp (RM12651)	This study	60	493
	reverse	TTCGTGTCCAATGGTGCTAA				
	forward	AGCTGAACTTGCATGGATGG	*C. volucris*	This study	60	298
	reverse	AAGTCCTTTAAGCTCTGCTCCA				
gluP	forward	AGCTCCACCCCAATAATAAGC	*C.* Sp (RM12651)	This study	60	604
	reverse	TCAAAATGGTGTTGTAGGTGGA				
hlyB	forward	TAGTTTGCTCTTGCGGTGTAT	*C.* Sp (RM12651)	This study	58	695
	reverse	CGCAAAATTAGGCAGTGAGT				
pgiB	forward	TGGATGATGTAAATGCAGTGCT	*C.* Sp (RM12651)	This study	60	662
	reverse	TGCGATTATATGTGARCCACCA				
gyrA T86I	forward	GCCCGTATAGTGGGTGCTGT	*C. jejuni*	Pholwat, S.[2]	60	86
	reverse	TCTTGAGCCATTCTAACCAAAGC	fluoroquinolone			
	probe^{W}	ATAAACTGCTGTATCTC	resistance			
	probe^{M}	AACTGCTATATCTCC				
23S	forward	GATCCAGTGAAATTGTAGTGGAGGT	*Campylobacter*	Pholwat, S.[2]	60	158
A2075G	reverse	GGCTCATATACAACTGGCGTCATA	macrolide			
	probe^{M}	GACGGAAAGACC	resistance			
	Probe^{W}	GACGGAGAGACCC				

[a] The original primers and probes were used as previously published if it was not indicated as "Modified" or "This study".

Reference

1. Linton, D.; Owen, R.J.; Stanley, J. Rapid identification by PCR of the genus Campylobacter and of five Campylobacter species enteropathogenic for man and animals. *Res Microbiol* **1996**, *147*, 707-718, doi:10.1016/s0923-2508(97)85118-2.
2. Pholwat, S.; Pongpan, T.; Chinli, R.; Rogawski McQuade, E.T.; Thaipisuttikul, I.; Ratanakorn, P.; Liu, J.; Taniuchi, M.; Houpt, E.R.; Foongladda, S. Antimicrobial Resistance in Swine Fecal Specimens Across Different Farm Management Systems. *Front Microbiol* **2020**, *11*, 1238, doi:10.3389/fmicb.2020.01238.
3. Dingle, K.E.; Colles, F.M.; Wareing, D.R.; Ure, R.; Fox, A.J.; Bolton, F.E.; Bootsma, H.J.; Willems, R.J.; Urwin, R.; Maiden, M.C. Multilocus sequence typing system for Campylobacter jejuni. *J Clin Microbiol* **2001**, *39*, 14-23, doi:10.1128/jcm.39.1.14-23.2001.
4. Miller, W.G.; On, S.L.; Wang, G.; Fontanoz, S.; Lastovica, A.J.; Mandrell, R.E. Extended multilocus sequence typing system for Campylobacter coli, C. lari, C. upsaliensis, and C. helveticus. *J Clin Microbiol* **2005**, *43*, 2315-2329, doi:10.1128/jcm.43.5.2315-2329.2005.
5. Hill, J.E.; Paccagnella, A.; Law, K.; Melito, P.L.; Woodward, D.L.; Price, L.; Leung, A.H.; Ng, L.K.; Hemmingsen, S.M.; Goh, S.H. Identification of Campylobacter spp. and discrimination from Helicobacter and Arcobacter spp. by direct sequencing of PCR-amplified cpn60 sequences and comparison to cpnDB, a chaperonin reference sequence database. *J Med Microbiol* **2006**, *55*, 393-399, doi:10.1099/jmm.0.46282-0.
6. Liu, J.; Gratz, J.; Amour, C.; Nshama, R.; Walongo, T.; Maro, A.; Mduma, E.; Platts-Mills, J.; Boisen, N.; Nataro, J.; et al. Optimization of Quantitative PCR Methods for Enteropathogen Detection. *PLoS One* **2016**, *11*, e0158199, doi:10.1371/journal.pone.0158199.
7. Poudel, S.; Li, T.; Chen, S.; Zhang, X.; Cheng, W.H.; Sukumaran, A.T.; Kiess, A.S.; Zhang, L. Prevalence, Antimicrobial Resistance, and Molecular Characterization of Campylobacter Isolated from Broilers and Broiler Meat Raised without Antibiotics. *Microbiol Spectr* **2022**, *10*, e0025122, doi:10.1128/spectrum.00251-22.

Table S2. Distribution of *Campylobacter* detected in different migratory bird species at 4 habitats.

Migratory birds<br>Order	Family	Genus	Hebei	Heilongjiang	Qinghai	Xizang	Total
Anseriformes	Anatidae	*Anas platyrhynchos*		2			2
		Anser albifrons/fabalis	1	31	1		33
		Anser anser/Anser erythropus			4	1	5
		Anser indicus	13		11	47	71
		Aythya nyroca			1		1
Charadriiformes	Laridae	*Chlidonias leucopterus*	1				1
		Chroicocephalus ridibundus	16			1	17
		Ichthyaetus relictus	1				1
		Larus argentatus	1				1
	Scolopacidae	*Numenius arquata*	2				2
		Tringa erythropus				1	1
	Recurvirostridae	*Recurvirostra avosetta*	2				2
	Charadriidae	*Charadrius placidus*	1				1
		Pluvialis squatarola	1				1
Suliformes	Phalacrocoracidae	*Phalacrocorax carbo*	4			1	5
Strigiformes	Strigidae	*Athene noctua*	1				1
Falconiformes	Falconidae	*Falco peregrinus*		1			1
	Total		44	34	17	51	146

Table S3. Detection of virulence genes in 5 *Campylobacter* species.

Virulence gene	Function	Detection Rate (%)*				
		C. jejuni	***C. coli***	***C. lari***	***C. volucris***	**RM12651-like**
cdtA	Exotoxin	76.5 (13/17)	66.7 (2/3)	81.8 (9/11)	75.0（3/4）	-
cdtB	Exotoxin	76.5 (13/17)	66.7 (2/3)	81.8 (9/11)	50.0（2/4）	-
cdtC	Exotoxin	23.5 (4/17)	33.3 (1/3)	81.8 (9/11)	50.0（2/4）	-
cadF	Adherence	70.6 (12/17)	33.3 (1/3)	91.7 (11/12)	100.0 （4/4）	84.3 (59/70)
cheY	Motility	86.7 (13/15)	66.7 (2/3)	91.7 (11/12)	100.0（4/4）	86.3 (63/73)
flaA	Motility	93.3 (14/15)	66.7 (2/3)	100.0 (12/12)	75.0（3/4）	91.8 (67/73)
ciaB	Invasion	80.0 (12/15)	100.0 (3/3)	83.3 (10/12)	100.0（4/4）	84.3 (59/70)
gluP	Immune modulation	-	-	-	-	75.7 (53/70)
pgiB	Immune modulation	-	-	-	-	78.1 (57/73)
hlyB	Exotoxin	-	-	-	-	72.6 (53/73)

*Due to the availability of the samples, no test was done on a few samples. Only samples with complete tests were included for Figure 2 and Figure 3B.

Supplemental Figure Legends

Figure S1. Phylogenetic analysis based on 16S rRNA gene in different habitats and migratory birds. Fasttree was used to construct the tree. *Helicobacter* was used as an outgroup. The figure was prepared with iTOL (Interactive Tree of Life). Different colors in the two columns represent different regions and birds, respectively. The Bootstrap value is displayed only when <0.7.

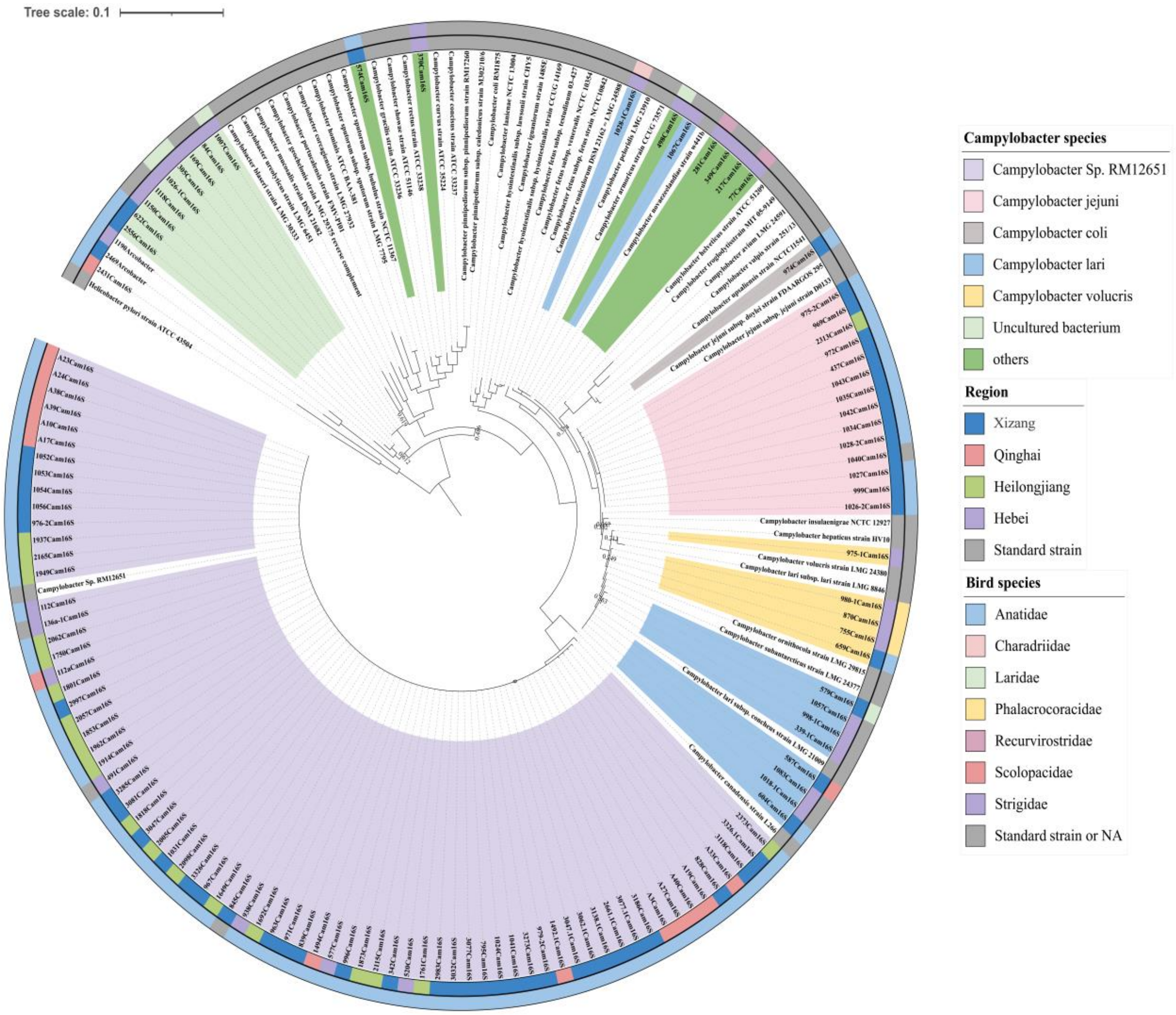
Tree scale: 0.1
Campylobacter species
Campylobacter Sp. RM12651
Campylobacter jejuni
Campylobacter coli
Campylobacter lari
Campylobacter volucris
Uncultured bacterium
others
Region
Xizang
Qinghai
Heilongjiang
Hebei
Standard strain
Bird species
Anatidae
Charadriidae
Laridae
Phalacrocoracidae
Recurvirostridae
Scolopacidae
Strigidae
Standard strain or NA
